# A microtubule-based minimal model for spontaneous and persistent spherical cell polarity

**DOI:** 10.1371/journal.pone.0184706

**Published:** 2017-09-20

**Authors:** Panayiotis Foteinopoulos, Bela M. Mulder

**Affiliations:** Systems Biophysics Department, Institute AMOLF, Amsterdam, the Netherlands; University of Illinois at Chicago, UNITED STATES

## Abstract

We propose a minimal model for the spontaneous and persistent generation of polarity in a spherical cell based on dynamic microtubules and a single mobile molecular component. This component, dubbed the polarity factor, binds to microtubules nucleated from a centrosome located in the center of the cell, is subsequently delivered to the cell membrane, where it diffuses until it unbinds. The only feedback mechanism we impose is that the residence time of the microtubules at the membrane increases with the local density of the polarity factor. We show analytically that this system supports a stable unipolar symmetry-broken state for a wide range of parameters. We validate the predictions of the model by 2D particle-based simulations. Our model provides a route towards the creation of polarity in a minimal cell-like environment using a biochemical reconstitution approach.

## Introduction

The establishment and maintenance of cell polarity, the spatially asymmetric distribution of intracellular components is of crucial importance to many developmental processes in biology, such as anisotropic growth morphologies and asymmetric divisions as precursors to differentiation. The unraveling of the subtle molecular mechanisms underlying these phenomena is an active field of biological research [1]. At the same time, the fundamental nature of this problem has also drawn the attention of biophysicists [2]. Building on Turing’s seminal work on biological pattern formation in reaction diffusion systems, the so-called Gierer-Meinhardt mechanism of a slow diffusing autocatalytic “activator” competing with a fast diffusing “inhibitor” has developed into a canonical modelling approach towards these questions (for a review see: [3]). The feasibility of such a mechanism was recently demonstrated by the Lim group [4], who designed such networks *in silico* and implemented them *in vivo* using a synthetic biology approach.

However, it appears that the cytoskeleton, the dynamic network of protein filaments that performs a host of structural and mechanical roles in all eukaryotic cells, is often implicated in polarity mechanisms [5]. A well-known example is fission yeast where microtubules are involved in depositing polarity factors to the cell ends, which in turn leads to the recruitment of actin nucleators, a key event in establishing polarized growth [6]. The question thus arises what role these non-diffusible filaments, whose primary role in interphase cells is to facilitate motor protein-driven linear transport, play in polarity generation. A class of polarity models proposed by the Altschuler-Wu group already implicitly includes the role of cytoskeletal filaments in the form of pre-positioned “patches” on the cell membrane in which the dynamics of a partially membrane bound target molecule is altered [7]. These models do indeed display persistent anisotropic patterns, yet, arguably, do not explain the spontaneous occurrence of symmetry-breaking, as the resultant patterns are predicated on the pre-established position of the patches. The same authors later also considered a single species self-activation model which does generate spontaneous symmetry breaking [8, 9]. However, in the latter model the patterns are not spatially persistent, but fluctuate over time, and disappear when the number of signalling molecules increases, indicating that this is an effect driven by finite particle number noise, rather than a steady state collective phenomenon. Recently, Freisinger et al. [10] presented the first quantitative model that addresses these shortcomings in the concrete setting of Cdc42 polarization in budding yeast. This model requires two feedback loops to yield a robust axis of polarization, one of which involves an actin-based Cdc42 recycling channel, which in turn is reinforced by actin nucleation stimulated by the presence of the active form of Cdc42. In this way the polarity factor Cdc42 can locally stabilize one of its delivery channels to the membrane, effectively spontaneously creating the “patches” of Ref. [7].

Here we show that the latter idea—positive feedback on membrane insertion through stabilisation of transporting structures—is *by itself* a sufficient mechanism to generate robust cell polarity. We do so by formulating a model that achieves the two desirable features of spontaneous symmetry breaking and steady-state persistence, using a minimal number of components. It is based on the proven ability of microtubules to bind and directionally transport proteins.

**Fig 1:**
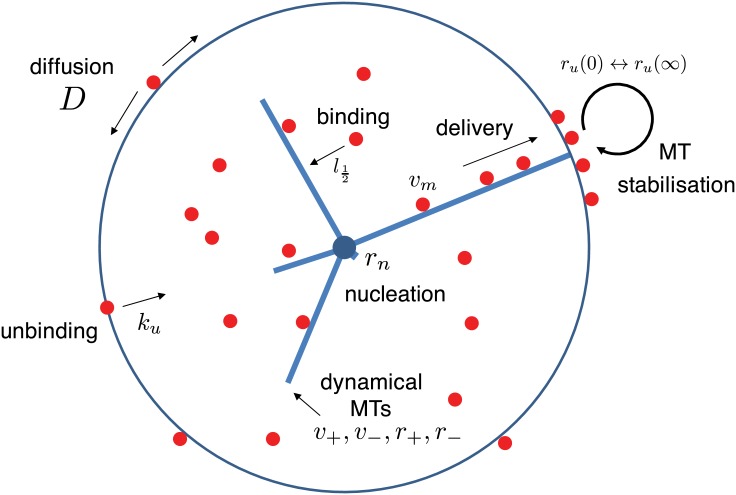
Schematic of the model. Dynamic microtubules transport polarity factors to the membrane. These are recycled to the cell interior after diffusing in and unbinding from the membrane. The polarity factors, however, stabilize microtubules against unbinding from the membrane, and thus are able to create local hotspots of polarity factor delivery creating a positive feedback loop leading to spontaneous polarization.

The key ingredient of the model is that the molecules acting as polarity factors, having been delivered to membrane by dynamical microtubules, stabilize the latter against detaching from the membrane. At the same time, a locally increased concentration of the polarity factors on the membrane depletes the finite pool of this species present in the cell providing a global inhibitory effect on the propensity of similar stable patches to develop elsewhere. Conceptually this model thus belongs to the generic class of activator-depletion models (for concrete examples see [11, 12] and [3] for a general overview), but distinguishes itself by employing the non-diffusible microtubules as a mediator species. Moreover, it allows an explicit analysis of the conditions under which polarization can occur. The model is schematically illustrated in Fig 1.

A somewhat similar model had been proposed earlier by Voituriez and coworkers [13, 14], albeit in a planar geometry. They considered cytoskeletal filaments nucleated from a membrane that are able to actively transport polarity factors from the cytosol towards the membrane, where they bind, diffuse and subsequently unbind. They implemented a feedback by letting the membrane density of nucleation points of filaments depend *linearly* on the local polarity factor density. Interestingly, they concluded that when the filaments are nucleated perpendicular to the membrane surface, which, as they suggest, corresponds to the radial spatial organization of microtubules we are considering here, polarization is in fact impossible. Only when the filaments were nucleated in an aster-like pattern, reminiscent of cortical actin organisation, was the in that case much more strongly enhanced local influx of polarity factors sufficient to generate polarization. The same conclusion was reached in a stochastic version of the Voituriez model [15]. We argue that the key difference in our model is the explicitly *non-linear* coupling between the polarity factors and the local density of microtubules, which is able to overcome the intrinsic limitations of effectively only 1d transport towards the membrane. The importance of non-linear competition effects for robust polarization is also stressed in the recent work on yeast polarity by Wu et al. [16]. It should also be noted that our model explicitly considers a full 3d geometry. The overwhelming majority of polarity models to date are 2d. An exception is the recent work of Klünder et al. [17] who considered a spherical model for Cdc42-driven yeast polarity.

## Results

### Model formulation

Our setting is a spherically symmetric cell of radius *R*, bounded by a membrane. Microtubules (MTs) are nucleated from a point-like centrosome in the cell center covered with a constant density of *m* nucleation sites per unit of solid angle, each of which can support a single MT. When unoccupied, these sites can “fire” with a rate *r*_*n*_, creating a new MT. The MTs obey the standard two-state dynamical instability model [18], with growth speed *v*_+_, shrinking speed *v*_−_, catastrophe rate *r*_+_ and rescue rate *r*_−_. When the microtubules hit the cell boundary they stall, after which they switch to the shrinking state with a rate $r_{u}(c_{b}(\hat{\omega },t))$ which depends on the local density $c_{b}(\hat{\omega },t)$ of the polarity factor (PF) in the membrane, where we use the unit vector $\hat{\omega }$ to parameterize the cell boundary. This dependence is described by

$$\begin{array}{c} r_{u}\left(c_{b}\right)=(r_{u}\left(0\right)-r_{u}\left(\infty \right))\sigma (\frac{c_{b}}{c_{*}})+r_{u}\left(\infty \right), \end{array}$$ (1)

The dose-response function *σ*, which interpolates the unbinding rate between the (higher) value *r*_*u*_(0) when no PFs are present and the (lower) saturation value *r*_*u*_(∞) depends on the reduced density *γ* ≡ *c*_*b*_/c_*_, where c_*_ sets the relevant density scale parameter. Although the specific choice for *σ* is not very critical (see S1 File), we adopt a simple standard sigmoidal type function

$$\begin{array}{c} \sigma (\gamma )=\frac{1}{1+\gamma^{p}},\;p>1, \end{array}$$ (2)

which introduces the Hill coefficient *p*.

The PFs in the cell interior freely diffuse and can bind to the MTs on a time scale much shorter than the MT dynamics. We therefore assume that they are in equilibrium with the instantaneous MT configuration, and their degree of binding only depends on the total length of MTs *l*_*tot*_ and a single affinity parameter $l_{\frac{1}{2}}$. Once bound to a MT they are transported to the MT plus end with speed *v*_*m*_, where they either delivered to the membrane, if the MT is in contact with the membrane, or simply fall off. Once in the membrane the PFs diffuse with (angular) diffusion constant *D* = *D*_*b*_/*R*^2^, until they unbind and are recycled into the interior of the cell with rate *k*_*u*_. The total number of PFs in our model thus is conserved and denoted by the parameter *C*. The mathematical details of this model can be found in the S1 File.

### Spontaneous polarization

In order to understand whether our model allows for spontaneous polarization, we study its steady-state behaviour. As it turns out, the steady state distribution of MT properties can be analytically determined for arbitrary distribution of PFs in the cell boundary. The details of all the relevant derivations can be found in S1 File. This explicit solution allows us to reduce the problem to a single autonomous reaction-diffusion equation for the PF density in the boundary

$$\begin{array}{c} D\Delta_{\hat{\omega }}c_{b}(\hat{\omega })-k_{u}c_{b}\left(\hat{\omega }\right)+K_{b}[c_{b}](\hat{\omega })=0, \end{array}$$ (3)

where the effective binding rate of PFs is given by

$$\begin{array}{c} K_{b}[c_{b}](\hat{\omega })=v_{m}c_{m}m_{b}(\hat{\omega }), \end{array}$$ (4)

i.e. proportional both to the density of MTs at the boundary $m_{b}\left(\hat{\omega }\right)$ and the number of PFs bound to MTs. One can show that this equation always admits an *isotropic* solution for any total number of PFs *C*. For details and the properties of this isotropic solution please refer to S1 File. To probe whether and if so, under which conditions, this equation also admits *anisotropic* solutions, we perform a standard stability analysis. We thus determine whether a small anisotropic perturbation $c_{b}^{\left(1\right)}\left(\hat{\omega }\right)$ to the isotropic background solution ${\bar{c}}_{b}$ can stably exist. To do so, this perturbation must satisfy the Helmholtz type wave equation

$$\begin{array}{c} \frac{D}{k_{u}}\Delta_{\hat{\omega }}c_{b}^{\left(1\right)}\left(\hat{\omega }\right)+\Omega^{2}\left({\bar{c}}_{b}/c_{*}\right)c_{b}^{\left(1\right)}\left(\hat{\omega }\right)=0, \end{array}$$ (5)

The squared wavenumber $\Omega^{2}({\bar{c}}_{b}/c_{*})$ is found to depend on a composite parameter

$$\begin{array}{c} \eta \equiv (\frac{r_{u}(\infty )}{r_{u}(0)}+\frac{\mu_{b}r_{n}}{\left(1+\mu_{i})r_{u}(0\right)})/(1-\frac{r_{u}(\infty )}{r_{u}(0)})>0, \end{array}$$ (6)

which is a decreasing function of the ratio *r*_*u*_(∞)/*r*_*u*_(0) and the number of MTs at the boundary, here represented by the dimensionless factor *μ*_*b*_.

At this point we have identified the four (effective) parameters that are involved in governing the propensity of the system to polarize.

1. The number of available polarity factors *C*, which in turn monotonically determines the relative mean membrane density $\gamma \equiv {\bar{c}}_{b}/c_{*},$ allowing us to adopt the latter as a direct measure of the availability of PFs and use at as the control parameter in the bifurcation problem.
2. The mean square angular displacement of PFs on the membrane before unbinding *δ* ≡ *D*/*k*_*u*_,
3. The Hill coefficient *p*, used in the definition of *σ*(*γ*), which governs the steepness of the dependency of the MT residence time on the PF density, and,
4. the composite parameter *η*, whose role we return to below.

A necessary requirement for Eq (5) to admit a solution is that $\Omega^{2}({\bar{c}}_{b}/c_{*})>0$. This requirement by itself already puts a number of constraints on the parameters:

$$\begin{array}{c} \eta <\eta_{max}=\frac{{\left(p-1\right)}^{2}}{4p} \end{array}$$ (7)

$$\begin{array}{c} \gamma >\gamma_{min}={\left(p-1\right)}^{-1/p} \end{array}$$ (8)

$$\begin{array}{c} p>p_{min}=1 \end{array}$$ (9)

The eigenfunctions in 3*d* of Eq (5) are known to be the spherical harmonics $Y_{n}^{m}\left(\hat{\omega }\right)$. Here, we are interested in the lowest, and most accessible, unipolar mode $Y_{1}^{0}\left(\hat{\omega }\right)=\cos\left(\theta \right)$ with angular momentum number *n* = 1. This mode can exist whenever

$$\begin{array}{c} \delta <\delta_{max}=\frac{1}{2}\left(p-1\right). \end{array}$$ (10)

**Fig 2:**
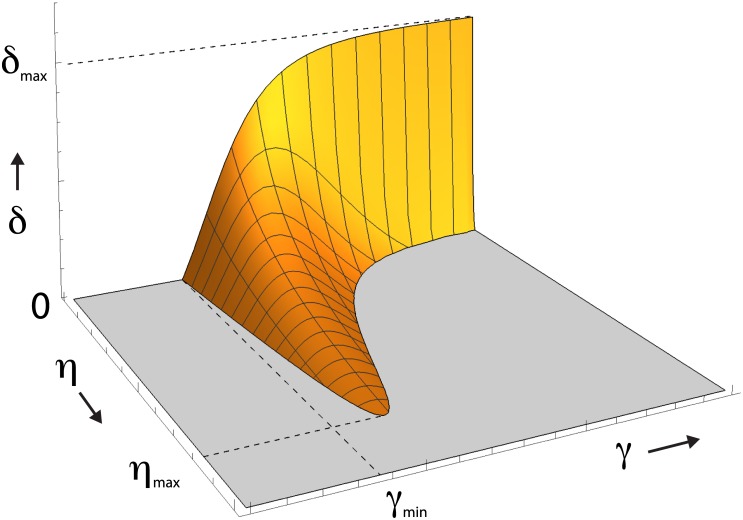
Phase diagram of the model. The polarized state is stable for the region of values of the parameters *η*, *γ* and *δ* enclosed by the depicted boundary surface, for the fixed value of the Hill coefficient *p* = 5.

The full phase boundary that separates the region where the unipolar solution is stable, from the one where only the isotropic solution can exists can be obtained by numerically solving $\Omega^{2}({\bar{c}}_{b}/c_{*})=2\delta$. The result in terms of the three parameters *η*, *γ* and *δ* for the case *p* = 5 is presented in Fig 2.

In summary, on the basis of these results, we can say that spontaneous polarization will occur whenever:

1. There is a sufficient number of PFs available (governed by increasing *γ*) to selectively promote the lifetime of MTs at the membrane.
2. The competitive advantage of MTs stabilized at the boundary is high enough (governed by decreasing *η*), because they stay much longer at the boundary than non-stabilized ones (smaller ratio *r*_*u*_(∞)/*r*_*u*_(0)) and/or the probability of reaching the boundary is small to begin with (smaller *μ*_*b*_) so that there are also fewer competitors for the finite supply of PFs.
3. The density dependence of the residence time enhancement of MTs is steep enough to have a more switch-like behaviour distinguishing the stabilized MTs from the non-stabilized ones (governed by increasing *p*).
4. After insertion the PFs unbind from the membrane before influencing other MTs at farther away locations —and hence in other directions— (governed by decreasing *δ*).

All these effects together promote the establishment of a localized stable polar patch of PFs, which then suppresses the formation of other such patches through the inhibitory pool depletion effect that decreases the availability of PFs to stabilize MTs at other locations.

**Fig 3:**
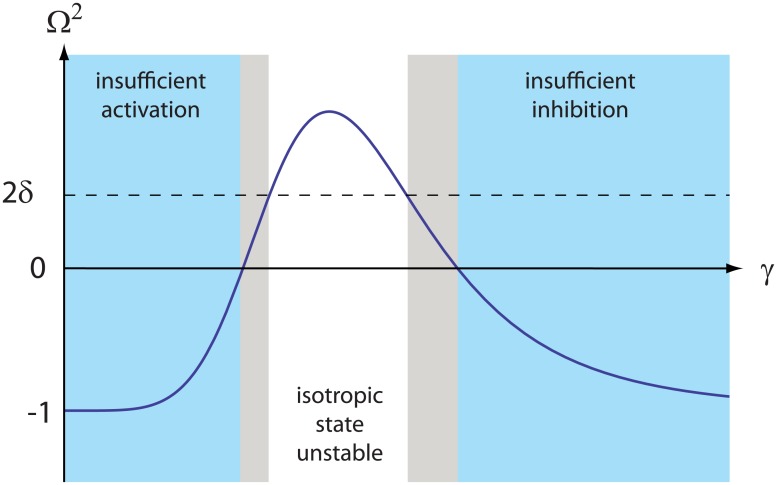
Polarization window. The angular wavenumber Ω^2^ as a function of the relative mean density of polarity factors in the membrane *γ*. Spontaneous polarization is possible only in the intermediate regime where the necessary condition Ω^2^ > 0 is fulfilled, and is achieved when the sufficient criterion Ω^2^ > 2*δ* is met (white area). In the other two regimes (blue areas) polarization is impossible due to insufficient activation (low *γ*) or insufficient inhibition (high *γ*).

For fixed values of *p*, *η* and *δ* which meet the criteria, a value of the total number of PFs *C* (through its proxy *γ*) can be found above which spontaneous symmetry breaking to a unipolar steady state occurs. However, as the pool of available PFs is increased, inevitably the polarization inducing mechanism breaks down: When the monotonically increasing average density of PFs in the membrane rises significantly above c_*_, the lifetime of all membrane-bound MTs becomes ≃ *r*_*u*_(∞)^−1^ independent of position, so that the differential stabilization mechanism is saturated and the system will revert back to the isotropic state. We thus expect that as a function of the number of available PFs we can distinguish three regimes: At low values of *γ* there are insufficient PFs bound to membrane to activate localized regions of longer-lived MTs. At high values of *γ*, the surfeit of available PFs precludes any localized increase of PFs to inhibit its accumulation at other locations, and MTs are equally stabilized everywhere. Only in the intermediate regime, where activation balances inhibition can sustained polarization be achieved. Fig 3 graphically illustrates this analysis, which also explains the reentrant behavior evident from the phase diagram, where at finite *η* and suitably small *δ* any line parallel to the *γ* axis pierces the ordered region at two locations.

In our model we have assumed that the amount of tubulin is not a limiting factor. However, since we are considering a finite volume, it is reasonable to ask to what extent our results would be influenced by potential finite tubulin pool size limitations. Indeed, recent experiments [19] have shown that the size of cytoskeletal structures, such as the mitotic spindle, could well be limited by tubulin availability. To address this question, we have also explicitly modelled the effect of a finite tubulin pool on the MT dynamics, specifically on the growth speed and the nucleation rate (see S1 File). For simplicity, we disregarded the effects of the capping of lengths due to cell boundary, focussing on the first order effects. The analysis shows that, due to a finite tubulin pool, the MTs are on average expected to be *shorter* than in the saturated case. This decreases the fraction of MTs reaching the boundary, and hence decreases the parameter *η* Eq (6), in fact *enhancing* the propensity to polarize. At the same time, however, the number of active microtubules decreases, but our model can be made robust against smaller MT numbers as shown by the analysis in S1 File (and explicitly validated by the simulations described below). We are therefore confident that our main conclusions are robust against finite pool size effects.

### Simulations

In order to validate the results of the theoretical model described above, and to test its viability in the light of known data on relevant cellular parameters we turn to simulations. We first note that the dimensional dependence of the model is in fact very weak, and essentially only enters through the eigenvalue of the angular laplacian (i.e. *n*^2^ in 2D vs. *n*(*n* + 1) in 3D). For simplicity’s sake, we therefore choose to provide proof-of-principle of our mechanism by simulations of a 2D stochastic version of our model in which both PFs and MTs are explicitly modelled as particles. Since the theoretical model is of a mean field nature, and implicitly assumes a continuous density of MTs, we first focus on a relatively large number of *M* = 10^3^ MTs.

All simulations are started from an initial state with no active MTs and all PFs localized to the cell interior. All measurements are performed after an equilibration phase, making sure the system has reached a steady state.

To measure the degree of polarization in the steady state we employ the polar order parameter

$$\begin{array}{c} S_{1}=\sqrt{{\left\langle \cos\theta \right\rangle }^{2}+{\left\langle \sin\theta \right\rangle }^{2}} \end{array}$$ (11)

where and *θ* is the angular position coordinate on the cell surface and the angular brackets denote ensemble averaging over the distribution of PFs in the cell boundary. This parameter takes on a value of 0 for a perfectly isotropic system and a value of 1 for a fully polarized system, where all PFs accumulate on a single spot.

**Fig 4:**
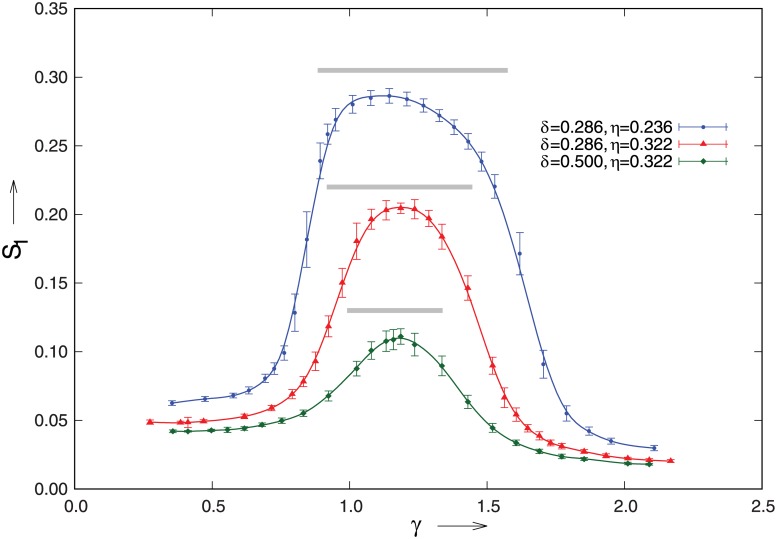
Comparison between theory and the simulations. The order parameter *S*_1_ as a function of the average membrane density of polarity factors *γ* as determined in 2*D* particle-based simulations with *p* = 5 and *R* = 3*μm*. The parameter *η* was tuned by changing the spontaneous catastrophe rate of the microtubules, yielding microtubules of mean length $\bar{l}=1.77\mu m$ (top curve) and $\bar{l}=2.54\mu m$ (middle and bottom curves).The corresponding theoretical predictions for the polarized regimes are shown as gray bars above each curve. Error bars denote standard errors in the mean from multiple independent runs.

To facilitate the comparison with the theory we convert the input parameter *C* into a corresponding value of the relative membrane density $\gamma ={\bar{c}}_{b}/c_{*}$, using a numerically obtained isotropic solution to Eq (3). In Fig 4 we present the results of the simulations for three different values of *η* and *δ*.

**Fig 5:**
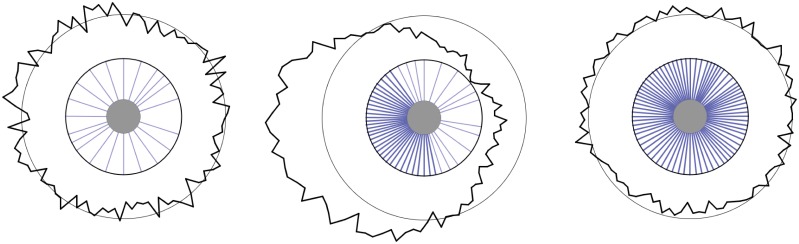
Snapshots of the simulation. Left: *γ* = 0.923, *C* = 30000, center: *γ* = 1.288, *C* = 37000, and right: *γ* = 1.853, *C* = 50000. The thick outer contour is a radial histogram of the polarity factor density at the boundary, with the outer circle marking the mean density level. The inner circle represents the cell boundary. For presentation purposes multiple microtubules resident at the boundary are lumped together, the pale blue lines representing a lower density and the dark blue lines a higher density. Also, all microtubules of length < *R* are not shown, and the dense central area is masked by the gray disk. Values of the remaining parameters: *η* = 0.322, *δ* = 0.286 and *p* = 5. Results show the predicted sequence of a low polarity factor membrane density isotropic state due to insufficient activation, an intermediate density polarized state, and a high-density state which is again unpolarized due to saturation.

These results show that the observed polarization is both qualitatively and quantitatively captured well by the theory, albeit that inevitable finite particle number effects shift the phenomenon to slightly higher values of *γ*. Fig 5 shows snapshots of the system in the low-*γ* isotropic, the intermediate polarized and the high-*γ* saturated regime respectively.

Arguably, the number of *M* = 10^3^ used here is large compared to a more realistic value of *M* ∼ 10^2^ observed for mammalian centrosomes [20], or that apply to *in vitro* reconstruction of centrosomes [21]. To understand whether our model is still able to achieve polarization in the latter case, we make use of the fact that both the total MT length *l*_*tot*_ and the density of MTs at the boundary $m_{b}\left(\hat{\omega }\right)$ are proportional to the total density of microtubules *m*. This implies that a scaling of the affinity parameter $l_{\frac{1}{2}}$ with *m* will leave the effective binding rate *K*_*b*_ (Eq (4)) invariant, and hence the solutions to the steady state Eq (3), invariant.

**Fig 6:**
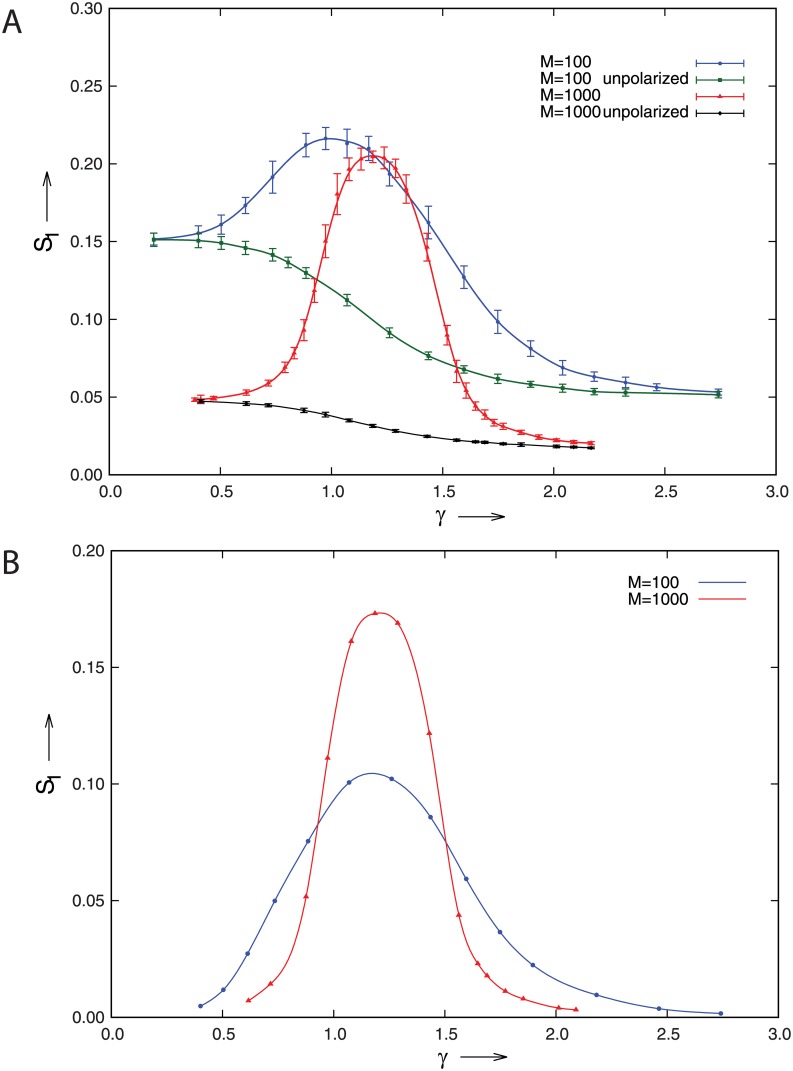
Effect of finite microtubule numbers. A) The order parameter *S*_1_ as a function of the average membrane density of polarity factors *γ* for the cases *M* = 100, *l*_1/2_ = 15*μm* and *M* = 1000, *l*_1/2_ = 150*μm*. Also shown are the “noise curves” obtained by artificially keeping the system in the unpolarized state. Error bars denote standard errors in the mean from multiple independent runs.B) The order parameter *S*_1_ as a function of the average membrane density of polarity factors, after noise subtraction.

This predicts that a decrease of the number of MTs *M* = 2*πm* can therefore be *exactly* compensated by a concomitant increased binding affinity of the PFs to the MTs. Using the observed values for *C*_*int*_ and *l*_*tot*_ in the simulation, in combination with the chosen parameter *l*_1/2_ = 150*μm*, we deduce that the so-called binding affinity (see Methods) is *v* ≃ 0.03. A comparison to the literature values for the Microtubule Associated Protein complex Dam1 [22], shows that we are still operating in a very low binding-density regime. This suggests that it is realistically feasible to compensate for a decrease in the number of MTs to a more realistic value of *M* = 10^2^, by a tenfold increase of the binding affinity (i.e. a tenfold decrease of $l_{\frac{1}{2}}$). To validate this prediction, we performed simulations with *M* = 100 and *l*_1/2_ = 15*μm*, comparing it to the case *M* = 1000 and *l*_1/2_ = 150*μm*. The results are shown in the top panel of Fig 6.

Although the peaks of the two curves appear to be fairly close, it is obvious that finite particle number effects are much more prominent at *M* = 100, as evidenced by the significantly higher values of *S*_1_ in the isotropic phase, be it in the regime of insufficient activation (∼factor of 3 larger than at *M* = 1000) or of insufficient inhibition (∼factor of 2 larger than at *M* = 1000). To estimate the magnitude of these effects we performed additional simulations in which we keep the total number of PFs in the membrane equal to that of the original simulations, but artificially maintain an isotropic unpolarized state by “homogenizing” the membrane density of PFs at each time step. This results in the lower curves in Fig 6A, which are in essence a lower-bound estimate of the finite particle number noise contribution to the observed degree of polarization. Subtracting these noise curves from the full results yields Fig 6B in which, as predicted, the location of the ordered peak is now seen to fully coincide between the two cases, leaving only a reduced amplitude and a slight broadening of the ordered region as the main effects of the reduced number of MTs.

## Methods

### Simulation details

We implement a 2*D* stochastic simulation in a circular cell geometry with a radius *R*. The control parameter in the simulations is the total number *C* of PFs in the system. A point-like centrosome in the center of the disc has *M* nucleation sites and the boundary of the disc is divided to *M* equal segments, each subtending an angle Δ*θ* = 2*π*/*M* around one of the nucleation directions. The number of PFs in the *m*’th segment is denoted by $C_{b}^{m}$. We define the local density of PFs as $c_{m}=(C_{b}^{m-1}+C_{b}^{m}+C_{b}^{m+1})/(3\Delta \theta ),$ i.e. we average over a neighbourhood also containing the flanking circle segments. This slightly dampens the potentially strong finite number of fluctuations at low values of *C*, an approximation reasonable in view of the specific parameters chosen in the simulation.

At the boundary, a PF can either diffuse or unbind thus recycling back into the cell interior. The probability of unbinding from the membrane in a single time step is given by *k*_*u*_Δ*t*, where Δ*t* is the time step and *k*_*u*_ is the unbinding rate. Correspondingly, the probability of diffusing on the membrane is given by 1 − *k*_*u*_Δ*t*. To determine the angular displacement *δθ* of a diffusing PF, we sample from the analytical form of the cumulative probability of diffusion on the unit circle

$$\begin{array}{c} P\left(\delta \theta \right)=\frac{\delta \theta }{2\pi }+\frac{1}{\pi }\sum_{n=1}\frac{e^{-n^{2}D\Delta t}}{n}\sin\left(n\delta \theta \right) \end{array}$$ (12)

As expected, the location of the maximum of the distribution (if this exists) can slowly drift over the unit circle. To obtain meaningful averages, we therefore corrected for this phase-drift, by extracting the phase through Fourier analysis and shifting the distribution accordingly.

Where available, we have used simulation parameters consistent with generic experimental values reported in the literature (see Table 1 [7, 22–31]).

**Table 1:** Model parameters.

Parameter	Simulation value	Reference value	Reference
R	3 *μ*m	(2.58±0.54)*μ*m	[23] Budding yeast
*v*_+_	0.013, 0.018 *μ*m/s	(0.010–0.033)*μ*m/s	[24] Budding yeast
*v*_−_	0.040, 0.045 *μ*m/s	(0.025–0.048)*μ*m/s	[24] Budding yeast
*r*_*n*_	0.05/s	(0.007–1.5)/*s*	[25] Budding Yeast,
			[26] Kidney epithelium
*r*_+_	0.0078/s	0.0078/s	[24] Budding Yeast
*r*_−_	0.0016/s	0.0016/s	[24] Budding Yeast
*v*_*m*_	0.81 *μ*m/s	(0.80–0.83)*μ*m/s	[27] Kinesin-1
*k*_*u*_	0.07/s	0.065/s	[28] Rac
D	0.02, 0.035 *μm*^2^/s	(0.036±0.017)/s	[7] Yeast
*M*	1000	60	[29] Xenopus egg
C	(0.10–0.80) × 10^5^	10^5^	[30] Eucaryotes
*l*_1/2_	15, 150 *μ*m	0–∞	[31], [22] Dam1 complex
2πc_*_	20 × 10^3^	unknown	

The binding affinity of PFs to MTs in our simulations is set by the parameter *l*_1/2_, while in the literature the “binding density” *v* is used as derived in the McGhee and von Hippel model [31] and defined for one-dimensional lattices as the number of moles of bound ligands per mole of total lattice residue.

To compare the two affinity parameters, we first consider the total number *N* of available dimers for binding, by introducing the length *l* = 8*nm* of a single tubulin dimer and taking into account that MTs have 13 protofilaments and find

$$\begin{array}{c} N=13\frac{l_{tot}}{l} \end{array}$$ (13)

The binding density (assuming each dimer can bind a PF) is then simply

$$\begin{array}{c} \nu =\frac{C_{m}}{N} \end{array}$$ (14)

with *C*_*m*_ the number of PFs bound to MTs. On the other hand, using the explicit definitions of the total MT length and the PF binding equilibrium discussed in the S1 File we find a relation between the number of MT-bound PFs, the total length of MT and *l*_1/2_

$$\begin{array}{c} l_{1/2}=\frac{C_{f}}{C_{m}}l_{tot}=\frac{C_{int}}{C_{m}}l_{tot}-l_{tot}, \end{array}$$ (15)

where *C*_*int*_ = *C*_*f*_ + *C*_*m*_ is the total number of PFs in the cell interior. Combining the two results, yields the desired relation between the two affinity parameters

$$\begin{array}{c} l_{1/2}=\frac{C_{int}l}{13v}-l_{tot} \end{array}$$ (16)

At each value of *C* a number of independent simulations were performed, allowing an error estimate of *S*_1_ to be obtained. For details on the statistics of the simulations please refer to S1 File.

## Discussion

We have presented a minimal, yet feasible, model for spontaneous cell polarization. Although it comprises no less than 13 parameters, our explicit analysis shows that its behavior is in fact only governed by 4 quantities, the *competition parameter* *η* (Eq (6)), which regulates the competitive advantage of stabilized MTs to recruit more stabilizing PFs, the *mean angular displacement* *δ* = *D*/*k*_*u*_ of PFs in the membrane, which determines the extent to which PFs once inserted remain localized, the *Hill parameter* *p*, which controls the steepness of the switch that distinguishes stabilized MTs from non-stabilized MTs at the membrane, and finally the *relative membrane density* $\gamma ={\bar{c}}_{b}/c,$ which controls the availability of PFs in the membrane to drive the polarization mechanism. We argue that the roles played by these 4 quantities are universal for a whole class of polarization mechanisms which rely on the autocatalytic enhancement of localized insertion, and as such transcend the specifics of the model presented here.

Although the model we propose does not correspond to any presently known polarity mechanism in vivo, it is fully based on feasible molecular roles. The role of MT-mediated transport in maintaining cell polarity is well established in fission yeast, where the polarity factors Tea1/Tea4 are transported through association with MT plus-end tracking (+TIPs) proteins such as Mal3 and the kinesin Tea2 to ensure the polar localization of cell growth [32]. Recently, Recouvreuz et al. [33] have “deconstructed” this system, by using a explicitly engineered chimeric complex using the membrane binding domain of Pom1 coupled to Mal3. This minimal system also displays clear polar enrichment. This also shows that using +TIPs, of which a large number have been identified through the work of Akhmanova and others [34, 35], is an alternative to more classical plus-end transporters such as the kinesin family of motor proteins [36]. Perhaps the most crucial part of our mechanism is the ability to stabilize MTs at the membrane. Here there is recent work that shows that Agrin mediates the localized capture of MTs and subsequent stabilization by Clasp2 at the synaptic membrane of neuromuscular cells [37]. Similarly, the actin binding protein Moesin has been shown to directly bind to MTs and stabilize them, albeit in the cortex and not at the membrane proper. On the whole we are therefore confident that our mechanism may, at the very least, form the basis of a biochemical reconstitution approach to set up polarity in a minimal cell-like environment, such as lipid bilayer-enclosed microvolumes containing purified and/or engineered protein components. Steps in this direction are currently actively pursued e.g. by the Dogterom lab [38, 39].

It is of course also interesting to consider how the current model can be coupled to polarized cell growth to further elucidate the biologically highly relevant interplay between cell shape, microtubule organisation and polarization.

## Supporting information

S1 File Supplementary information. (PDF)
