# Supplementary material for: A microtubule-based minimal model for spontaneous and persistent spherical cell polarity
Source: PLoS One. 2017 Sep 20;12(9):e0184706. doi: 10.1371/journal.pone.0184706 (PMC5607169; doi:10.1371/journal.pone.0184706)
Supplement: S1 File — (PDF) [file pone.0184706.s001.pdf]

Supplementary Material to "A Microtubule-based Minimal Model for Spontaneous and Persistent Spherical Cell Polarity"

Panayiotis Foteinopoulos and Bela M. Mulder

FOM Institute AMOLF, Science Park 104,

1098XG Amsterdam, the Netherlands

CONTENTS

I. Dynamical equations 2

II. Steady state 4

III. Bifurcation analysis 7

IV. Properties of the isotropic solution 8

V. Requirements on the dose-response function $\sigma(\gamma)$ 9

VI. Effect of a finite tubulin pool 10

A. Dynamical equations 10

B. Steady state solution 12

VII. Simulation Statistics 14

References 14

I. DYNAMICAL EQUATIONS

The dynamical equations for the MTs in our model are given by

$$\partial_t m_i^+(l,\hat{\omega},t) = -v_+\partial_l m_i^+(l,\hat{\omega},t) + r_- m_i^-(l,\hat{\omega},t) - r_+ m_i^+(l,\hat{\omega},t), \tag{1}$$

$$\partial_t m_i^-(l,\hat{\omega},t) = v_-\partial_l m_i^-(l,\hat{\omega},t) - r_- m_i^-(l,\hat{\omega},t) + r_+ m_i^+(l,\hat{\omega},t), \tag{2}$$

$$\partial_t m_0(\hat{\omega},t) = -r_n m_0(\hat{\omega},t) + v_- m_i^-(l=0,\hat{\omega},t), \tag{3}$$

$$\partial_t m_b(\hat{\omega},t) = -r_u\left(c_b(\hat{\omega},t)\right) m_b(\hat{\omega},t) + v_+ m_i^+(R,\hat{\omega},t), \tag{4}$$

where $m_i^+(l,\hat{\omega},t)$ and $m_i^-(l,\hat{\omega},t)$ are the densities of growing and shrinking MTs in the direction $\hat{\omega}$ in the cell interior, $m_b(\hat{\omega},t)$ the density of stalled MTs at the boundary, and $m_0(\hat{\omega},t)$ the density of unoccupied nucleation sites, conveniently considered as density of "dormant" MTs. These equations are supplemented by the boundary conditions

$$r_n m_0(\hat{\omega}, t) = v_+ m_i^+(l = 0, \hat{\omega}, t), \tag{5}$$

$$r_u\left(c_b(\hat{\omega}, t)\right) m_b(\hat{\omega}, t) = v_- m_i^-(R, \hat{\omega}, t), \tag{6}$$

and the MT conservation law

$$m = m_0(\hat{\omega}, t) + \int_0^R dl\ \left\{ m_i^+(l, \hat{\omega}, t) + m_i^-(l, \hat{\omega}, t) \right\} + m_b(\hat{\omega}, t). \tag{7}$$

The PF can either be adsorbed to the membrane, on which it diffuses with (angular) diffusion constant $D = D_b/R^2$ and from which it unbinds with rate k_u, freely diffuse within the interior of the cell, where we assume it diffuses with effective diffusion constant $D_i = \infty$ or bind to MTs. Its conservation law reads

$$C = C_f(t) + C_m(t) + \int d\hat{\omega}\, c_b(\hat{\omega}, t), \tag{8}$$

where C_f is the number of free PFs in the interior, C_m the number bound to MTs and c_b the density on the membrane. We assume that the binding kinetics of the PF to the MTs is fast with respect to the other processes, so it can be described as an equilibrium, with the density (per unit length) of PFs on MTs given by

$$c_m(t) = \frac{C_m(t)}{l_{tot}(t)} = \frac{1}{l_{tot}(t) + l_{\frac{1}{2}}} \left(C - \int d\hat{\omega}\, c_b(\hat{\omega}, t) \right), \tag{9}$$

where

$$l_{tot}(t) = \int d\hat{\omega} \int_0^R dl\, l \left\{ m_i^+(l, \hat{\omega}, t) + m_i^-(l, \hat{\omega}, t) \right\} + R \int d\hat{\omega}\, m_b(\hat{\omega}, t) \tag{10}$$

is the total length of all MTs in the system, and $l_{\frac{1}{2}}$ is a parameter that sets the affinity of the PFs for binding to the MTs. The PFs bound to the MTs are transported towards the MT plus end with speed v_m, where they are delivered to the membrane, if the MT they are bound to is attached to the membrane, or they simply fall off. The time evolution of the PF density on the membrane is thus described by

$$\partial_t c_b(\hat{\omega}, t) = D \Delta_{\hat{\omega}} c_b(\hat{\omega}, t) - k_u c_b(\hat{\omega}, t) + v_m c_m(t)\, m_b(\hat{\omega}, t), \tag{11}$$

where $\Delta_{\hat{\omega}}$ is the Laplacian on the unit sphere. The final ingredient of the model we need to specify is the dependency of the residence time of the MTs at the boundary on the local density of PFs, which we parametrize as

$$r_u(c_b) = (r_u(0) - r_u(\infty))\,\sigma\left(\frac{c_b}{c_*}\right) + r_u(\infty), \tag{12}$$

Here σ is a dose-response function, which depends on the reduced density $\gamma \equiv c_b/c_*$, where c_* is defines the relevant density scale parameter. In principle the only properties we require of $\sigma(\gamma)$ is to monotonically decrease from its maximal value $\sigma(0) = 1$ and to vanish sufficiently fast for large arguments, i.e. $\lim_{\gamma\to\infty} \gamma\sigma(\gamma) = 0$ (see Section V). For concreteness sake, however, we will in the following adopt a standard sigmoidal Hill type function

$$\sigma(\gamma) = \frac{1}{1+\gamma^p}, \quad p > 1. \tag{13}$$

II. STEADY STATE

In this section we derive the steady state solutions to the MT dynamical equations. The steady state equations for the MTs are given by

$$0 = -v_+\partial_l m_i^+(l,\hat{\omega}) + r_- m_i^-(l,\hat{\omega}) - r_+ m_i^+(l,\hat{\omega}), \tag{14}$$

$$0 = v_-\partial_l m_i^-(l,\hat{\omega}) - r_- m_i^-(l,\hat{\omega}) + r_+ m_i^+(l,\hat{\omega}), \tag{15}$$

$$0 = -r_n m_0(\hat{\omega}) + v_- m_i^-(l=0,\hat{\omega}), \tag{16}$$

$$0 = -r_u\left(c_b(\hat{\omega})\right) m_b(\hat{\omega}) + v_+ m_i^+(R,\hat{\omega}), \tag{17}$$

with boundary conditions

$$r_n m_0(\hat{\omega}) = v_+ m_i^+(l=0,\hat{\omega}), \tag{18}$$

$$r_u\left(c_b(\hat{\omega})\right) m_b(\hat{\omega}) = v_- m_i^-(R,\hat{\omega}). \tag{19}$$

Combining Eqs. (14), (15), (17) and (19) leads to the well-known [1] balance law

$$v_+ m_i^+(l,\hat{\omega}) = v_- m_i^-(l,\hat{\omega}) \tag{20}$$

for all $l \in [0, R]$. This allows the elimination of $m_i^-(l, \hat{\omega})$ in Eq. (14) and its solution with the aid of Eq. (16):

$$m_i^+(l, \hat{\omega}) = \frac{r_n}{v_+} m_0(\hat{\omega}) e^{-l/\bar{l}}, \tag{21}$$

$$m_i^-(l, \hat{\omega}) = \frac{r_n}{v_-} m_0(\hat{\omega}) e^{-l/\bar{l}}, \tag{22}$$

where

$$\bar{l} = \left(\frac{r_c}{v_+} - \frac{r_r}{v_-}\right)^{-1} \tag{23}$$

is the mean MT length in the absence of boundaries. We can now use the MT conservation law and the boundary condition Eq. (19) which here reads

$$r_u\left(c_b(\hat{\omega})\right) m_b(\hat{\omega}) = v_- m_i^-(R, \hat{\omega}) = v_+ m_i^+(R, \hat{\omega}) = r_n m_0\left(\hat{\omega}\right) e^{-R/\bar{l}} \tag{24}$$

to determine

$$m_b\left(\hat{\omega}\right) = \frac{m r_n e^{-R/\bar{l}}}{\left(\left(1 + r_n \bar{l}\left(\frac{1}{v_+} + \frac{1}{v_-}\right)\left(1 - e^{-R/\bar{l}}\right)\right) r_u\left(c_b(\hat{\omega})\right) + r_n e^{-R/\bar{l}}\right)}, \tag{25}$$

$$m_0\left(\hat{\omega}\right) = \frac{m r_u\left(c_b(\hat{\omega})\right)}{\left(\left(1 + r_n \bar{l}\left(\frac{1}{v_+} + \frac{1}{v_-}\right)\left(1 - e^{-R/\bar{l}}\right)\right) r_u\left(c_b(\hat{\omega})\right) + r_n e^{-R/\bar{l}}\right)}. \tag{26}$$

This shows that in steady state all MT densities are fully determined by the density of PFs at the membrane. For convenience, we now introduce the following dimensionless quantities

$$\mu_i \equiv r_n \bar{l}\left(\frac{1}{v_+} + \frac{1}{v_-}\right)\left(1 - e^{-R/\bar{l}}\right), \tag{27}$$

$$\mu_b \equiv e^{-R/\bar{l}}, \tag{28}$$

which respectively are proportional to the number of MTs of length $< R$, corresponding to the cell interior, and of length R, thus reaching the boundary, *in the absence of boundary effects*, i.e. dependent only on the MT dynamical parameters. We now turn to the explicit calculation of the total length of the MTs (Eq. (10)), which works out as

$$l_{tot}\left[c_b\right] = m\bar{l} \int d\hat{\omega} \frac{r_n \bar{l}\left(\frac{1}{v_+} + \frac{1}{v_-}\right)\left(1 - e^{-R/\bar{l}}\left(1 + \frac{R}{\bar{l}}\right)\right) r_u\left(c_b(\hat{\omega})\right) + \frac{R}{\bar{l}} r_n e^{-R/\bar{l}}}{\left(\left(1 + r_n \bar{l}\left(\frac{1}{v_+} + \frac{1}{v_-}\right)\left(1 - e^{-R/\bar{l}}\right)\right) r_u\left(c_b(\hat{\omega})\right) + r_n e^{-R/\bar{l}}\right)}.$$

We introduce the dimensionless quantities

$$\lambda_i \equiv r_n \bar{l}\left(\frac{1}{v_+} + \frac{1}{v_-}\right)\left(1 - e^{-R/\bar{l}}\left(1 + \frac{R}{\bar{l}}\right)\right), \tag{29}$$

$$\lambda_b \equiv \frac{R}{\bar{l}} e^{-R/\bar{l}}, \tag{30}$$

proportional to the mean length stored in MTs of length $< R$ and length equal to R respectively, again *in the absence of boundary effects*. These definitions allow us to concisely write

$$l_{tot}\left[c_b\right] = m\bar{l}\int d\hat{\omega}\frac{\lambda_i r_u\left(c_b\left(\hat{\omega}\right)\right)+\lambda_b r_n}{\left(1+\mu_i\right)r_u\left(c_b\left(\hat{\omega}\right)\right)+\mu_b r_n} \tag{31}$$

and

$$m_b\left(\hat{\omega}\right) = m\frac{r_n\mu_b}{\left(1+\mu_i\right)r_u\left(c_b(\hat{\omega})\right)+\mu_b r_n}. \tag{32}$$

So, in steady state both the density c_m of PFs on the MTs and the density $m_b(\hat{\omega})$ of MTs at the boundary functionally depend on the density $c_b(\hat{\omega})$ of PFs in the membrane. This allows us to formulate a single autonomous equation for the latter density

$$D\Delta_{\hat{\omega}}c_b\left(\hat{\omega}\right) - k_u c_b(\hat{\omega}) + K_b\left[c_b\right]\left(\hat{\omega}\right) = 0, \tag{33}$$

where, combining Eq. (9) and the steady state solutions, the effective binding rate K_b of PFs to membrane is given by

$$\begin{aligned} K_b\left[c_b\right]\left(\hat{\omega}\right) \equiv & v_m c_m m_b\left(\hat{\omega}\right) \\ = & \frac{m v_m}{l_{tot}\left[c_b\right]+l_{\frac{1}{2}}}\left(C-\int d\hat{\omega}' c_b\left(\hat{\omega}'\right)\right)\frac{\mu_b r_n}{\left(1+\mu_i\right)r_u\left(c_b\left(\hat{\omega}\right)\right)+\mu_b r_n}. \end{aligned} \tag{34}$$

We now note that since l_tot is proportional to the overall MT density (Eq. (31)), K_b is invariant under the scaling $m \to \alpha m$ and $l_{\frac{1}{2}} \to \alpha l_{\frac{1}{2}}$. This implies that any change in overall MT density can be exactly compensated by an correspondingly increased affinity of PFs to bind to MTs, which leaves the density of PFs in the membrane, and hence the behaviour of the system as a whole, unchanged.

Clearly, Eq. (33) admits an isotropic solution $c_b\left(\hat{\omega}\right) = \bar{c}_b\left(C\right)$ for any value of the number of PFs. This solution obeys

$$-k_u\bar{c}_b + K_b(C,\bar{c}_b) = 0. \tag{35}$$

While not soluble on closed form, we can prove the property that $\bar{c}_b\left(C\right)$ is monotonically increasing with C and is asymptotically linear, $\bar{c}_b\left(C\right) \propto C,\ C \to \infty$ (neglecting saturation of binding), so that $\bar{c}_b\left(C\right)$ can take on any positive value (see SI Section IV), a property which we will need below.

III. BIFURCATION ANALYSIS

We now ask whether Eq. (33) can also support an anisotropic solution. To that end we adopt a standard bifurcation approach and consider the first order response of the isotropic solution to a perturbation of the type $c_b(C) = \bar{c}_b(C) + \varepsilon\, c_b^{(1)}(\hat{\omega})$ where $\varepsilon \ll 1$. This leads to the Helmholtz equation

$$\frac{D}{k_u}\Delta_{\hat{\omega}} c_b^{(1)}(\hat{\omega}) + \Omega^2(\bar{c}_b/c_*)\, c_b^{(1)}(\hat{\omega}) = 0, \tag{36}$$

where the square of the dimensionless wavenumber is given by

$$\Omega^2(\bar{c}_b/c_*) = -\left(1 + \frac{\bar{c}_b}{c_*}\frac{\sigma'(\bar{c}_b/c_*)}{\sigma(\bar{c}_b/c_*) + \eta}\right), \tag{37}$$

and

$$\eta \equiv \left(\frac{r_u(\infty)}{r_u(0)} + \frac{\mu_b r_n}{(1+\mu_i)\, r_u(0)}\right) / \left(1 - \frac{r_u(\infty)}{r_u(0)}\right) > 0. \tag{38}$$

As the spherical harmonics $Y_n^m(\hat{\omega})$ are eigenfunctions of the spherical Laplacian, they are also solutions to the bifurcation equation (36) provided $\Omega^2(\gamma) = n(n+1)\,\delta$. Provided there is a value of the relative membrane PF density for which $\Omega^2(\gamma) > 0$, a suitably small value of the angular displacement parameter δ can be found, such that the unipolar ($n = 1$) solution $Y_1^0(\hat{\omega}) \propto \cos\theta$ is accessible. Given that $\Omega^2(0) = -1$ by definition, we thus need to ensure that $\Omega^2(\gamma)$ has zeros. Explicitly

$$\Omega^2(\gamma) = \frac{p\gamma^p}{(\gamma^p + 1)(\eta + \eta\gamma^p + 1)} - 1, \tag{39}$$

so that $\Omega^2(\gamma) = 0$ leads to quadratic in γ^p which has two real solutions provided

$$\eta < \eta_{\max} = (p-1)^2/4p. \tag{40}$$

Importantly, this shows that there is an upper bound condition on the parameter η. Note that η can be made arbitrarily small by both *increasing* the residence time of the MTs at the boundary, i.e. reducing $r_u(\infty)$, while simultaneously *decreasing* the probability of MTs reaching the boundary, either through reducing the nucleation rate r_n or decreasing the mean MT length $\bar{l}$, which exponentially suppresses the factor μ_b (cf. Eq. (27)). This analysis suggests that the smaller the parameter η, the more competitive advantage a MT stabilized at the boundary has; either because it stays much longer at the membrane allowing it to deposit yet more PFs, and/or because it has fewer MTs in other orientations competing for

stabilization. Using Eq. (39) we can also determine the maximum value of $\Omega^2(\gamma)$, which is given by

$$\max \Omega^2(\gamma) = p - 1 - 2p\left(\sqrt{\eta(1+\eta)} - \eta\right) \leq p - 1. \tag{41}$$

Unipolar solutions to the bifurcation equation can therefore only exist provided (recall that $n = 1$)

$$2\delta < 2\delta_{\min} = p - 1. \tag{42}$$

This requirement can readily be interpreted: if a PF released by a stabilized MT travels too far along the membrane before unbinding, it may contribute to the stabilization of competing MTs in other directions, thus decreasing the propensity for polarization. Finally, explicitly solving $\Omega^2(\gamma) = 0$ in the limit $\eta \downarrow 0$, yields an absolute lower bound on the number of PFs necessary to achieve polarization, which is given by $\gamma_{\min} = (p-1)^{-1/p}$. Such a bound is expected, since a minimal density of PFs is required to achieve stabilization of MTs in the first place. Note that the bounds on all these three parameters provide an *a posteriori* explanation of the requirement on the Hill coefficient $p > p_{\min} = 1$.

IV. PROPERTIES OF THE ISOTROPIC SOLUTION

Here we derive the fact that the density of PFs on the membrane in the isotropic state increases monotonically with the total number of PFs C, which is a minimal requirement for the bifurcation to the polarized state to occur.

The explicit form for the effective binding rate in the isotropic state that appears in Eq. (35) is

$$K_b(C, \bar{c}_b) = \frac{M v_m}{l_{tot}(\bar{c}_b) + l_{\frac{1}{2}}} (C - 4\pi\bar{c}_b) \frac{\mu_b r_n}{(1+\mu_i) r_u(\bar{c}_b) + \mu_b r_n}. \tag{43}$$

with

$$l_{tot}(\bar{c}_b) = 4\pi M \bar{l} \frac{\lambda_i r_u(\bar{c}_b) + \lambda_b r_n}{(1+\mu_i) r_u(\bar{c}_b) + \mu_b r_n}. \tag{44}$$

Trivially, $\bar{c}_b(C=0) = 0$, and an explicit calculation shows that

$$\frac{d}{dC}\bar{c}_b(C=0) = \frac{v_m \bar{m}_b(\bar{c}_b = 0)}{k_u\left(l_{tot}(\bar{c}_b = 0) + l_{\frac{1}{2}}\right) + 4\pi v_m \bar{m}_b(\bar{c}_b = 0)} \geq 0. \tag{45}$$

Taking the derivative of Eq. (35) with respect to the total number of PFs C and rearranging, yields

$$\frac{d}{dC}\bar{c}_b(C) = \frac{\frac{\partial}{\partial C} K_b(C, \bar{c}_b(C))}{k_u - \frac{\partial}{\partial \bar{c}_b} K_b(C, \bar{c}_b(C))}. \tag{46}$$

We now note that, from Eq. (43)

$$\frac{\partial}{\partial C}K_b\left(C,\bar{c}_b\left(C\right)\right)=\frac{K_b\left(C,\bar{c}_b\left(C\right)\right)}{C-4\pi\bar{c}_b\left(C\right)}>0. \tag{47}$$

Thus $\frac{d}{dC}\bar{c}_b\left(C\right)$ can only change sign through a pole (ruled out as $K_b\left(C,\bar{c}_b\right)$ is bounded) or a cusp (ruled out by smoothness of $K_b\left(C,\bar{c}_b\right)$), showing that $\bar{c}_b\left(C\right)$ is monotonically increasing.

Finally, the form of Eq. (43) and the asymptotic behaviour of $r_u\left(\bar{c}_b\right)=r_u\left(\infty\right)+\mathcal{O}\left(\left(\bar{c}_b\right)^{-1-\varepsilon}\right)$ as $\bar{c}_b\to\infty$, which follows from the Ansatz $\lim_{x\to\infty}x\sigma\left(x\right)=0$ on the dose-response function, dictate that $\bar{c}_b\left(C\right)$ is asymptotically linear. An explicit calculation gives

$$\bar{c}_b\left(C\right)\sim\frac{v_m\bar{m}_b\left(\bar{c}_b=\infty\right)}{k_u\left(l_{tot}\left(\bar{c}_b=\infty\right)+l_{\frac{1}{2}}\right)+4\pi v_m\bar{m}_b\left(\bar{c}_b=\infty\right)}C,\ C\to\infty. \tag{48}$$

Together these results imply that $\bar{c}_b\left(C\right)$ can take on any positive value by suitably choosing C.

V. REQUIREMENTS ON THE DOSE-RESPONSE FUNCTION $\sigma\left(\gamma\right)$

Here we derive the minimal requirements the dose-response function $\sigma\left(\gamma=\bar{c}_b/c_*\right)$ must meet in order for polarization to be possible.

A necessary requirement for the bifurcation to the polar state to occur is that $\Omega^2(\gamma)>0$. This implies the requirement

$$S\left(\gamma\right)\equiv-\gamma\sigma'\left(\gamma\right)-\sigma\left(\gamma\right)=-\left(\gamma\sigma\left(\gamma\right)\right)'>\eta>0. \tag{49}$$

This criterion is met (for suitably small η) if $S\left(\gamma\right)$ has at least one zero for a finite value of γ. We first note that by definition $S\left(0\right)=-1$. Next consider

$$I_\infty\equiv\lim_{\gamma\to\infty}\int_0^\gamma d\gamma' S\left(\gamma'\right)=-\lim_{\gamma\to\infty}\gamma\sigma\left(\gamma\right)\leq 0. \tag{50}$$

By imposing the requirement $I_\infty=0$, i.e. $\lim_{\gamma\to\infty}\gamma\sigma\left(\gamma\right)=0$, we guarantee that $S\left(\gamma\right)>0$ for some $\gamma\in\left(0,\infty\right)$, so that it has at least one zero. If we furthermore assume that $\sigma\left(\gamma\right)$ has no more than a single inflection point on $(0,\infty)$, the zero of $S\left(\gamma\right)$ is moreover unique. Finally, under this same requirement we have that $\lim_{\gamma\to\infty}\Omega^2(\gamma)=-1$, and hence $\Omega^2(\gamma)$ only has has a single positive maximum.

This analysis also shows that the response function need not be sigmoidal. E.g. $\sigma\left(\gamma\right)=\exp\left(-\gamma\right)$ also works, as $S\left(\gamma\right)=\left(\gamma-1\right)\exp\left(-\gamma\right)$ has an obvious single zero at $\gamma=1$. In this case $\eta_{\max}=\max_\gamma S\left(\gamma\right)=\exp\left(-2\right)\simeq 0.135$.

VI. EFFECT OF A FINITE TUBULIN POOL

In our model we have so far assumed that the amount of tubulin is not a limiting factor. Since, however, we are working in a finite volume it is a reasonable question to ask to what extent our results are robust against possible finite tubulin pool size limitations. Here we address this question, by explicitly modelling the effect a finite pool has on the MT dynamics, specifically the growth speed and the nucleation rate. For simplicity, we disregard the effects of the capping of lengths due to cell boundary, focussing on the first order effects.

A. Dynamical equations

We assume the cell contains a finite amount of tubulin, which is represented in terms of a total microtubule length L_{tot}. There are M nucleation sites from which MTs can be nucleated with a rate given by

$$r_n\left(L_{free}\right) = r_n \frac{L_{free}^q}{L_{free}^q + L_{\frac{1}{2}}} \tag{51}$$

where L_{free} is the available free (non-polymerized) tubulin length, $L_{\frac{1}{2}}$ a cross-over parameter, which distinguishes the regime of low availability, in which the rate is strongly limited by the available free pool size, and the regime of high availability, in which the rate ultimately becomes independent of the free pool size. The Hill coefficient $q > 1$, describes the potential cooperativity necessary in the nucleation a new microtubule. Once nucleated, microtubules grow with a speed

$$v_+\left(L_{free}\right) = v_+ \frac{L_{free}}{L_{free} + L_{\frac{1}{2}}} \tag{52}$$

which at low availability captures the linear dependence of growth on the free tubulin density. The other dynamical parameters v_-, r_+ and r_- are considered to be *in*dependent of the available amount of free tubulin. Denoting by $M_0\left(t\right)$ the total number of free nucleation sites (orientation is unimportant in this context), the dynamics of the system is described

by the equations

$$\frac{d}{dt}M_0\left(t\right) = -r_n\left(L_{free}\left(t\right)\right)M_0 + v_-m_-\left(0,t\right) \tag{53}$$

$$\frac{\partial}{\partial t}m_+\left(l,t\right) = -v_+\left(L_{free}\left(t\right)\right)\frac{\partial}{\partial l}m_+\left(l,t\right) - r_+m_+\left(l,t\right) + r_-m_-\left(l,t\right) \tag{54}$$

$$\frac{\partial}{\partial t}m_-\left(l,t\right) = v_-\frac{\partial}{\partial l}m_+\left(l,t\right) - r_+m_+\left(l,t\right) + r_-m_-\left(l,t\right) \tag{55}$$

$$\frac{d}{dt}L_{free}\left(t\right) = -v_+\left(L_{free}\left(t\right)\right)\int_0^\infty dlm_+\left(l,t\right) + v_-\int_0^\infty dlm_-\left(l,t\right) \tag{56}$$

with boundary condition

$$r_n\left(L_{free}\left(t\right)\right)M_0 = v_+\left(L_{free}\left(t\right)\right)m_+\left(0,t\right) \tag{57}$$

As a check on these equations we note that they should obey the conservation laws

$$M_0\left(t\right) + \int_0^\infty dl\left\{m_+\left(l,t\right) + m_-\left(l,t\right)\right\} = M \tag{58}$$

$$L_{free} + \int_0^\infty dll\left\{m_+\left(l,t\right) + m_-\left(l,t\right)\right\} = L_{tot} \tag{59}$$

Now note that

$$\int_0^\infty dl\left\{\frac{\partial}{\partial t}m_+\left(l,t\right) + \frac{\partial}{\partial t}m_-\left(l,t\right)\right\} = \int_0^\infty dl\left\{-v_+\left(L_{free}\left(t\right)\right)\frac{\partial}{\partial l}m_+\left(l,t\right) + v_-\frac{\partial}{\partial l}m_+\left(l,t\right)\right\} \tag{60}$$

$$= v_+\left(L_{free}\left(t\right)\right)m_+\left(0,t\right) - v_-m_-\left(0,t\right) = -\frac{d}{dt}M_0\left(t\right) \tag{61}$$

and

$$\int_0^\infty dll\left\{\frac{\partial}{\partial t}m_+\left(l,t\right) + \frac{\partial}{\partial t}m_-\left(l,t\right)\right\} = \int_0^\infty dll\left\{-v_+\left(L_{free}\left(t\right)\right)\frac{\partial}{\partial l}m_+\left(l,t\right) + v_-\frac{\partial}{\partial l}m_+\left(l,t\right)\right\} \tag{62}$$

$$= v_+\left(L_{free}\left(t\right)\right)\int_0^\infty dlm_+\left(l,t\right) - v_-\int_0^\infty dlm_-\left(l,t\right) = -\frac{d}{dt}L_{free}\left(t\right) \tag{63}$$

where we have assumed $\lim_{l\to\infty} m_+\left(l,t\right) = \lim_{l\to\infty} m_-\left(l,t\right) = 0$.

B. Steady state solution

We now consider the steady state, for which

$$-r_n(L_{free})M_0 + v_- m_-(0) = 0 \tag{64}$$

$$-v_+(L_{free})\frac{\partial}{\partial l}m_+(l) - r_+ m_+(l) + r_- m_-(l) = 0 \tag{65}$$

$$v_-\frac{\partial}{\partial l}m_+(l) - r_+ m_+(l) + r_- m_-(l) = 0 \tag{66}$$

$$-v_+(L_{free})\int_0^\infty dl m_+(l) + v_-\int_0^\infty dl m_-(l) = 0 \tag{67}$$

with boundary condition

$$r_n(L_{free})M_0 = v_+(L_{free})m_+(0) \tag{68}$$

We note that Eqs. (65) and (66) lead to

$$m_+(l) = \frac{m_0}{v_+(L_{free})}e^{-l/\bar{l}} \tag{69}$$

$$m_-(l) = \frac{m_0}{v_-}e^{-l/\bar{l}} \tag{70}$$

where

$$\bar{l}(L_{free}) = \left(\frac{r_+}{v_+(L_{free})} - \frac{r_-}{v_-}\right)^{-1} \tag{71}$$

We can determine M_0, m_0 and L_{free} through the identities

$$M = M_0 + m_0\left(\frac{1}{v_+(L_{free})} + \frac{1}{v_-}\right)\bar{l}(L_{free}) \tag{72}$$

$$L = L_{free} + m_0\left(\frac{1}{v_+(L_{free})} + \frac{1}{v_-}\right)\bar{l}(L_{free})^2 \tag{73}$$

$$r_n(L_{free})M_0 = m_0 \tag{74}$$

so that

$$M_0 = \frac{M}{1 + r_n(L_{free})\left(\frac{1}{v_+(L_{free})} + \frac{1}{v_-}\right)\bar{l}(L_{free})} \tag{75}$$

$$L_{free} = L - \frac{r_n(L_{free})M}{1 + r_n(L_{free})\left(\frac{1}{v_+(L_{free})} + \frac{1}{v_-}\right)\bar{l}(L_{free})}\left(\frac{1}{v_+(L_{free})} + \frac{1}{v_-}\right)\bar{l}(L_{free})^2 \tag{76}$$

$$= L - \frac{r_n(L_{free})\left(\frac{1}{v_+(L_{free})} + \frac{1}{v_-}\right)\bar{l}(L_{free})}{1 + r_n(L_{free})\left(\frac{1}{v_+(L_{free})} + \frac{1}{v_-}\right)\bar{l}(L_{free})}M\bar{l}(L_{free}) \tag{77}$$

We now take as our length scale $l_0 = L_{\frac{1}{2}}/M$ and introduce $\lambda = L/L_{\frac{1}{2}}$, $\lambda_{free} = L_{free}/L_{\frac{1}{2}}$ and $\bar{\lambda}(\lambda_{free}) = \bar{l}(L_{free})/l_0$ so that

$$\lambda_{free} = \lambda - \frac{r_n(\lambda_{free})\left(\frac{1}{v_+(\lambda_{free})} + \frac{1}{v_-}\right)\bar{\lambda}(\lambda_{free})}{1 + r_n(\lambda_{free})\left(\frac{1}{v_+(\lambda_{free})} + \frac{1}{v_-}\right)\bar{\lambda}(\lambda_{free})}\bar{\lambda}(\lambda_{free}) \tag{78}$$

and note that

$$\frac{L_{free}}{L_{free} + L_{\frac{1}{2}}} = \frac{\lambda_{free}}{\lambda_{free} + 1} \tag{79}$$

Below, we now prove the following two inequalities:

1. $\bar{\lambda}(\lambda_{free}(\lambda)) \leq \bar{\lambda}(\lambda_{free}(\infty))$

2. $M - M_0(\lambda_{free}(\lambda)) \leq M - M_0(\lambda_{free}(\infty))$

The first inequality implies that, due to the finite tubulin pool, the MTs are on average *shorter* than in the saturated case. This decreases the fraction of MTs reaching the boundary, and hence decreases the parameter η (Main text, Eq. (40)), which in turn enhances the propensity to polarize. The second inequality implies that the average number of active MTs *decreases* due to the finite tubulin pool. However, as we show in the Main text (Section Simulations) the model can in fact be made robust against this decrease.

We first note that $r_n(\lambda_{free})$, $\left(\frac{1}{v_+(\lambda_{free})} + \frac{1}{v_-}\right)$ and $\bar{\lambda}(\lambda_{free})$ are all bounded as $\lambda_{free} \to \infty$, so that $\lambda_{free}(\lambda) \sim \lambda$ as $\lambda \to \infty$. As $r_n(0)$ and $\left(\frac{1}{v_+(\lambda_{free})} + \frac{1}{v_-}\right)\bar{\lambda}(\lambda_{free}) \to 1/r_+$ as $\lambda_{free} \downarrow 0$, we have $\lambda_{free}(0) = 0$. As the second term on the right hand side of Eq. (78) is definite positive, we conclude $\lambda_{free}(\lambda) \leq \lambda$, for all λ. Consider

$$\bar{\lambda}(\lambda) = \frac{M}{L_{\frac{1}{2}}}\left(\frac{r_+}{v_+}\frac{1+\lambda}{\lambda} - \frac{r_-}{v_-}\right)^{-1} \tag{80}$$

Thus $\bar{\lambda}(0) = 0$ and $\bar{\lambda}(\infty) < \infty$.

Moreover,

$$\frac{d}{d\lambda}\bar{\lambda}(\lambda) = \frac{M}{L_{\frac{1}{2}}}\left(\frac{r_+}{v_+}\frac{1+\lambda}{\lambda} - \frac{r_-}{v_-}\right)^{-2}\frac{r_+}{v_+}\frac{1}{\lambda^2} \geq 0 \tag{81}$$

so that $\bar{\lambda}(\lambda_{free}(\lambda)) \leq \bar{\lambda}(\lambda) \leq \bar{\lambda}(\infty) = \bar{\lambda}(\lambda_{free}(\infty)) < \infty$. Likewise,

$$\frac{d}{d\lambda}\left\{\left(\frac{1}{v_+(\lambda)} + \frac{1}{v_-}\right)\bar{\lambda}(\lambda)\right\} = \frac{M}{L_{1/2}}\frac{r_+ + r_-}{((r_-v_+ - r_+v_-)\lambda - r_+v_-)^2} \geq 0 \tag{82}$$

and

$$\frac{d}{d\lambda} r_n(\lambda) = \frac{q r_n \lambda^{q-1}}{(\lambda^q + 1)^2} \geq 0 \tag{83}$$

So, we can write

$$\frac{d}{d\lambda} \left\{ r_n(\lambda) \left(\frac{1}{v_+(\lambda)} + \frac{1}{v_-} \right) \bar{\lambda}(\lambda) \right\} \geq 0 \tag{84}$$

We also have $r_n(\infty) \left(\frac{1}{v_+(\infty)} + \frac{1}{v_-} \right) \bar{\lambda}(\infty) < \infty$, and so

$$\frac{M - M_0(\lambda_{free}(\lambda))}{M} \leq \frac{M - M_0(\lambda)}{M} = \frac{r_n(\lambda) \left(\frac{1}{v_+(\lambda)} + \frac{1}{v_-} \right) \bar{\lambda}(\lambda)}{1 + r_n(\lambda) \left(\frac{1}{v_+(\lambda)} + \frac{1}{v_-} \right) \bar{\lambda}(\lambda)}$$
$$\leq \frac{M - M_0(\infty)}{M} = \frac{M - M_0(\lambda_{free}(\infty))}{M} \tag{85}$$

VII. SIMULATION STATISTICS

To calculate the order parameter S_1 for each value of the control parameter C, we performed several runs and then we averaged over them. The number of runs that was executed for each C is included in Tables I through VI.

C(x10^3)	15	20	24	26	28	29	30	31	32	33	33.5	34	35	36
Runs	20	20	20	20	20	20	20	20	20	20	20	20	60	40
C(x10^3)	37	38	39	40	41	42	43	44	46	48	50	52	54	58
Runs	70	50	90	90	80	110	80	40	50	20	20	20	20	20

TABLE I. Number of simulation runs for different values of the control parameter C for the case $\delta = 0.286$, $\eta = 0.236$, $M = 1000$.

[1] Marileen Dogterom and Stanislas Leibler. Physical aspects of the growth and regulation of microtubule structures. *Physical review letters*, 70(9):1347–1350, 1993.

C(x10^3)	14	17	22	25	27	28	29	30	31	32	33	34	35	36
Runs	10	20	20	20	30	10	10	20	20	20	40	20	40	40

C(x10^3)	37	38	40	42	43	44	45	46	47	48	50	52	54	56
Runs	40	10	20	20	30	20	30	20	30	20	40	20	10	20

TABLE II. Number of simulation runs for different values of the control parameter C for the case $\delta = 0.286$, $\eta = 0.322$, $M = 1000$.

C(x10^3)	13	15	18	20	22	24	26	28	30	32	33	34
Runs	10	20	20	20	30	10	10	20	20	20	40	20

C(x10^3)	34.5	35	36	38	40	42	44	46	48	50	54	56
Runs	10	20	20	30	20	30	20	30	20	40	20	10

TABLE III. Number of simulation runs for different values of the control parameter C for the case $\delta = 0.500$, $\eta = 0.322$, $M = 1000$.

C(x10^3)	3.65	7.30	14.59	18.24	21.88	25.53	29.18	31.00	32.83	34.65
Runs	20	20	20	20	20	10	30	20	40	30

C(x10^3)	36.48	40.12	43.77	47.42	51.06	54.71	58.36	62.01	65.66	72.95
Runs	30	50	40	40	90	100	80	65	60	20

TABLE IV. Number of simulation runs for different values of the control parameter C for the case $\delta = 0.286$, $\eta = 0.322$, $M = 100$.

C(x10^3)	3.65	7.30	14.59	18.24	21.88	25.53	27.35	29.18	32.83
Runs	30	30	50	80	70	80	70	70	60

C(x10^3)	36.48	40.12	43.77	47.42	51.06	54.71	58.36	62.01	72.95
Runs	30	40	40	50	60	80	90	70	30

TABLE V. Number of simulation runs for different values of the control parameter C for the case $\delta = 0.286$, $\eta = 0.322$, $M = 100$, constant c_b.

C(x10^3)	10	15	22	25	29	31	33	35	37	40	43	45	46	48	50	54	56	58
Runs	30	20	20	20	40	40	20	40	20	20	40	20	20	20	20	20	20	20

TABLE VI. Number of simulation runs for different values of the control parameter C for the case $\delta = 0.286$, $\eta = 0.322$, $M = 1000$, constant c_b.
